# Machine Learning-Enhanced Extraction of Biomarkers for High-Grade Serous Ovarian Cancer from Proteomics Data

**DOI:** 10.1038/s41597-024-03536-1

**Published:** 2024-06-25

**Authors:** Senuri De Silva, Asfa Alli-Shaik, Jayantha Gunaratne

**Affiliations:** 1 Institute of Molecular and Cell Biology (IMCB), Agency for Science, Technology and Research (A*STAR), Singapore, 138673 Singapore; 2 Yong Loo Lin School of Medicine, National University of Singapore, Singapore, 117594 Singapore

**Keywords:** Proteome informatics, Cancer screening

## Abstract

Comprehensive biomedical proteomic datasets are accumulating exponentially, warranting robust analytics to deconvolute them for identifying novel biological insights. Here, we report a strategic machine learning (ML)-based feature extraction workflow that was applied to unveil high-performing protein markers for high-grade serous ovarian carcinoma (HGSOC) from publicly available ovarian cancer tissue and serum proteomics datasets. Diagnosis of HGSOC, an aggressive form of ovarian cancer, currently relies on diagnostic methods based on tissue biopsy and/or non-specific biomarkers such as the cancer antigen 125 (CA125) and human epididymis protein 4 (HE4). Our newly developed ML-based approach enabled the identification of new serum proteomic biomarkers for HGSOC. The performance verification of these marker combinations using two independent cohorts affirmed their outperformance against known biomarkers for ovarian cancer including clinically used serum markers with >97% AUC. Our analysis also added novel biological insights such as enriched cancer-related processes associated with HGSOC.

## Introduction

Mass spectrometry (MS)-based proteomics has significantly expanded its applications in clinical translational research^1,2^. This powerful technology offers unbiased identification and quantification of thousands of proteins within biological samples, rendering it well-suited for system-wide investigation of disease functional mechanisms and identification of potential biomarkers. The accumulation of such MS-based high-quality proteomics data in the public domain and deep analysis of these datasets by advanced analytic pipelines, including strategic machine learning (ML) approaches, can yield additional yet important biological insights.

Advancements in artificial intelligence approaches such as deep learning and ML have led to their widespread use in various fields. By utilizing these analytics technologies, it is possible to learn from data adjusting to sample size limitations and data variability without the reliance on predefined thresholds. These methods with systematically customized workflows are ideal for unveiling highly discriminating features from complex proteomics datasets of a wide variety of diseases including highly heterogenous diseases such as cancer.

High-grade serous ovarian carcinoma (HGSOC) is a highly malignant ovarian cancer that comprises high mortality rates with a five-year survival rate of 34%^3,4^. Detecting HGSOC is challenging due to its asymptomatic nature and rapid disease progression. Accurate detection typically requires a biopsy, which is a highly invasive procedure associated with several complications such as bleeding and infections^5^. Current HGSOC detection approaches rely on a collection of clinically utilized serum biomarkers that are used for ovarian cancer in general, namely CA125 (carbohydrate antigen 125 or mucin-16) and HE4 (human epididymis protein 4 or WAP four-disulfide core domain protein 2). Ovarian cancer diagnostic tests such as the risk of malignancy index based on CA125 levels, ovary imaging, and risk of ovarian malignancy algorithm with serum biomarkers CA125 and HE4 alongside the menopausal status lack specific detection of HGSOC^6–8^. These reasons underscore the urgent need for novel, high-performance circulating markers that can be utilized independently or synergistically to stratify patients with HGSOC in a non-invasive fashion.

Here, we show the identification of novel serum biomarkers and biological insights pertaining to HGSOC through reanalysis of two publicly available HGSOC proteomics datasets^9,10^ utilizing a strategic ML-based feature extraction pipeline. From these high-quality datasets comprising 153 participants, we systematically characterized proteome changes in ovarian tissue and serum through stand-alone and integrative analyses and strategized a ML workflow to identify and high performing HGSOC-specific biomarkers dysregulated in both serum and tissue. Finally, we identified high-performing serum biomarker panels for HGSOC and benchmarked its predictive capability alongside established clinical tests.

## Results

### Tissue and serum proteome dynamics in HGSOC

**Fig. 1:**
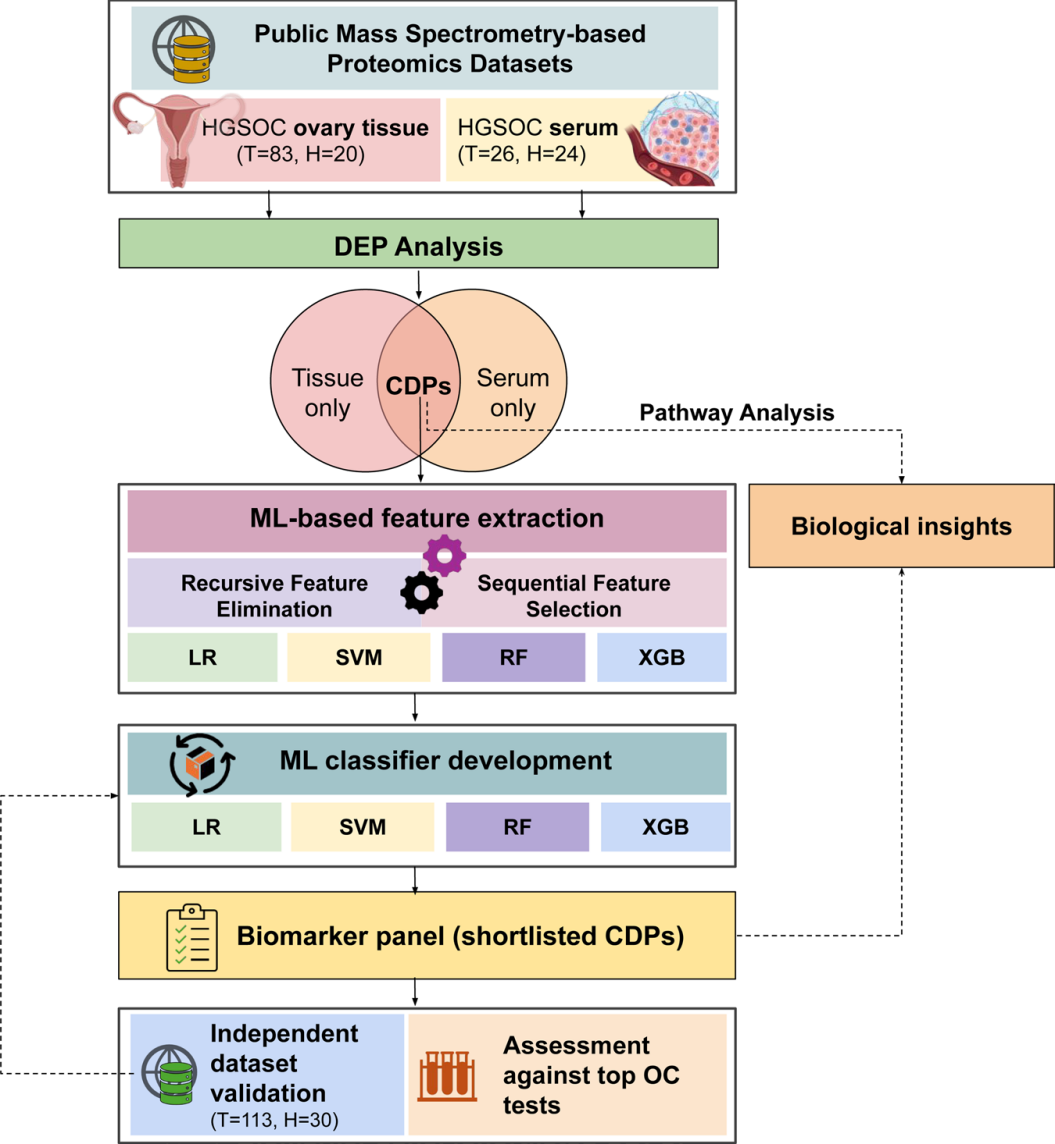
Machine learning-based biomarker extraction workflow for HGSOC. Publicly available mass spectrometry-based serum and ovary tissue proteomics data from 109 HGSOC and 44 healthy controls were utilized as input data. The differential expression protein (DEP) analysis was conducted separately on tissue and serum to capture HGSOC related protein signatures. Then commonly dysregulated proteins (CDPs) across tissue and serum were identified and their biological insights were extracted through pathway analysis. Machine learning (ML) based feature extraction was carried out to shortlist the most discriminant signatures found in the serum with recursive feature extraction and sequential feature selection method utilizing logistic regression (LR), support vector machine (SVM), random forest (RF), and extreme gradient boosting (XGB) algorithms. ML models were built to discriminate between HGSOC and healthy with the identified signatures from the previous step employing LR, SVM, RF and XGB algorithms as classifiers. The biological insights of the biomarkers were investigated through literature review. Independent publicly available datasets were used to verify the marker performances. The diagnostic capability of the signatures was compared with seven best-in-class ovarian cancer (OC) tests (Clipart from BioRender.com).

**Fig. 2:**
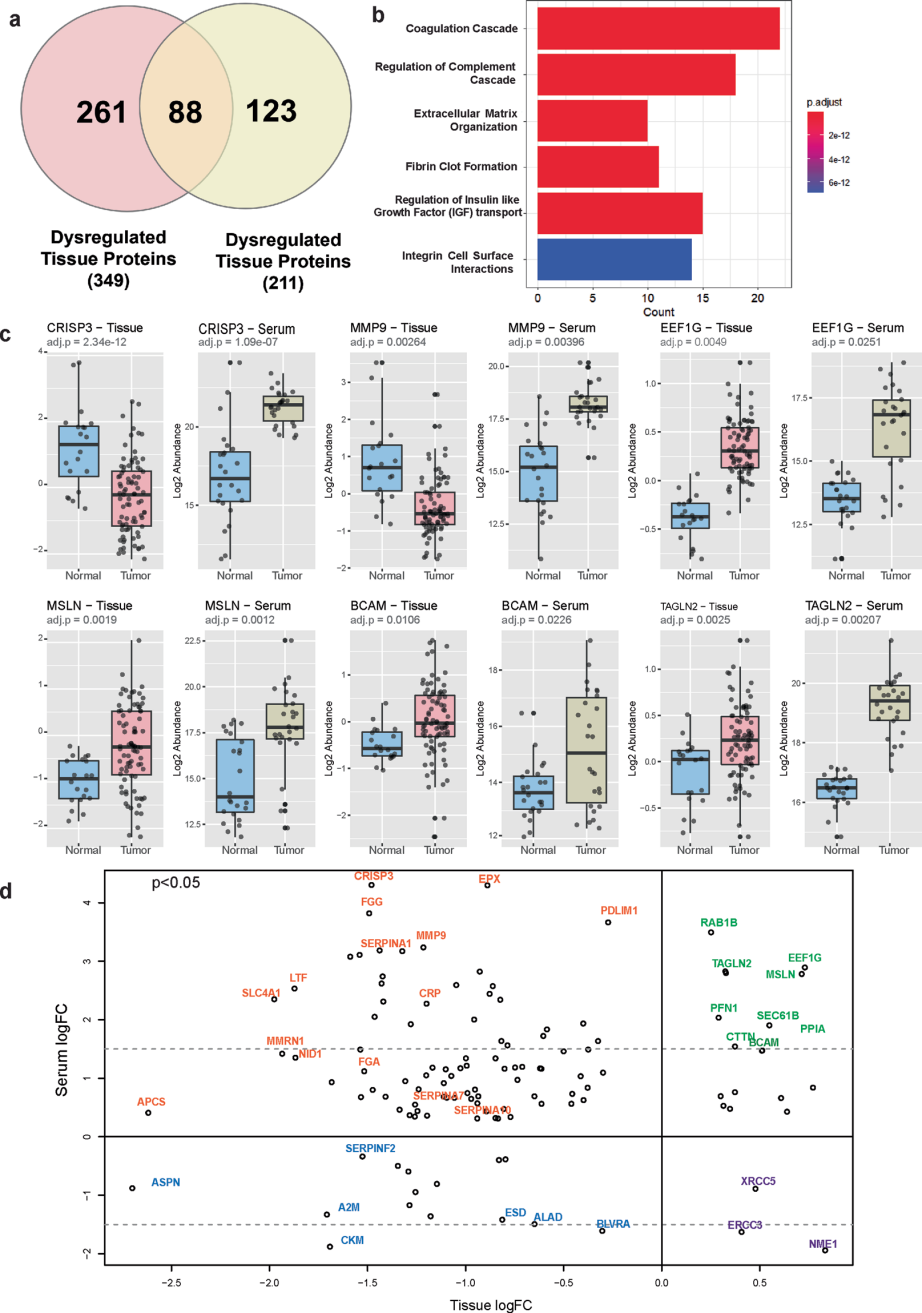
Co-dysregulation of biopsy and serum ovarian cancer proteome. (**a**) Venn diagram of the dysregulated proteins in ovary tissue and serum. (**b**) Bar plot of the pathway ontology terms (FDR-corrected P < 0.01) of significant CDPs. The horizontal axis represents the number of enriched genes in the pathway and the color of the bar plot highlights the adjusted p value. (**c**) The boxplots of the protein abundance differences of tumor vs normal samples in top six CDPs in tissue (left) and serum (right) (FDR-corrected P < 0.05). (**d**) The log fold change (LFC)-based similarity analysis of serum and ovary tissue CDPs. The serum LFC is plotted as a function of the tissue LFC. Four clusters of proteins are separated by the LFC characteristics with colored dots and texts as follows; Green: upregulated in both serum and tissue; Blue: downregulated in both serum and tissue; Orange: upregulated in the serum but downregulated in tissue; Purple: downregulated in the serum but upregulated in the tissue.

The primary objective of this study was to uncover novel biological insights from publicly available datasets using strategized analytic approaches including a rigorous ML exercise (Fig. 1). For this, we used two published quantitative proteomics datasets from ovary tissue^9^ and serum^10^ analyses of 109 HGSOC patients in comparison to 44 healthy controls. First, we extracted co-dysregulated proteins (CDPs) among differentially expressed proteins (DEPs) in tissue and serum obtained by originally reported statistical criteria. We identified 88 CDPs that exhibit significant dysregulations in both datasets (Fig. 2a, Supplementary Table 1).

Gene ontology enrichment analysis of CDPs showed significant enrichment of coagulation cascade (C8B, CFHR3, CFI, CFP, CPB2, CPN2, CRP, MASP1, PROS1) (Supplementary Fig. 1a), and intrinsic pathway of fibrin clot formation (F13A1, KNG1, MMRN1, ORM1, PLG, PROS1). In addition, CDPs were found to be enriched in the extracellular matrix (ECM) organization (COL6A2, COL6A6, KLKB1, LAMB2, LTBP1, LUM, MMP9, NID1, CRISP3, PLG, TIMP2, TNXB, VWF, CPN2) (Supplementary Fig. 1b), and integrin cell surface interactions (COL6A2, COL6A6, LUM, VWF) (Fig. 2b, Supplementary Fig. 1b, Supplementary Table 2). It was notable that the ECM-related proteins were downregulated in the tissue and upregulated in the serum (Fig. 2c, Supplementary Fig. 2). The downregulation of ECM remodeling in tissue with fibrous proteins and proteoglycans in ovarian cancer is known to promote tumor progression^11–13^.

A log fold change (LFC)-based similarity analysis of CDPs was carried out to further study their interrelationships in ovary tissue and serum. Plotting the serum LFC as a function of tissue LFC showed four different clusters (Fig. 2d) of CDPs with intriguing patterns. The first cluster (Fig. 2d highlighted in green) consists of the CDPs with upregulation in both serum and tissue. These proteins were enriched in protein binding and more specifically cadherin binding while localizing into extracellular exosome and cytosol. This cluster encompassed a collection of oncogenic markers currently employed as diagnostic and prognostic biomarkers in cancer. For example, PFN1 is found to play an important role in tumor invasion and migration in endometrial^14^ and breast cancer^15^. Moreover, TAGLN2 and MSLN are characterized by their role in metastasis in the biliary tract and ovarian cancer^16–18^.

The second cluster comprised proteins with upregulation in the serum, but downregulation in the tissue (Fig. 2d highlighted in orange). The majority of the CDPs belong to this cluster although it has a negative correlation. For example, CRISP3 is a significantly characterized protein in this cluster with high alterations in both serum and tissue. This may suggest that CRISP3 might be actively released into the bloodstream from the tumor or surrounding tissues which has a potential diagnostic value. Previously reported serum diagnostic markers for epithelial ovarian cancer (EOC), such as CRP, are also included in this cluster^19^. Furthermore, oncogenic markers such as EPX, FGG, and PDLM1 are also part of this CDP cluster. Additionally, this cluster comprises many proteins from the serpin family. Serpin proteins are known cancer markers in colorectal and ovarian cancer^20–22^. The third and fourth clusters (Fig. 2d highlighted in blue and purple) included proteins downregulated in the serum which may provide interesting functional mechanisms of HGSOC. These downregulated proteins were enriched in platelet activation, cell-matrix adhesion, and inflammatory response and involved the pathways including neutrophil extracellular trap formation and ECM-receptor interaction.

### ML-based extraction of high performing serum biomarker combinations from CDPs

**Fig. 3:**
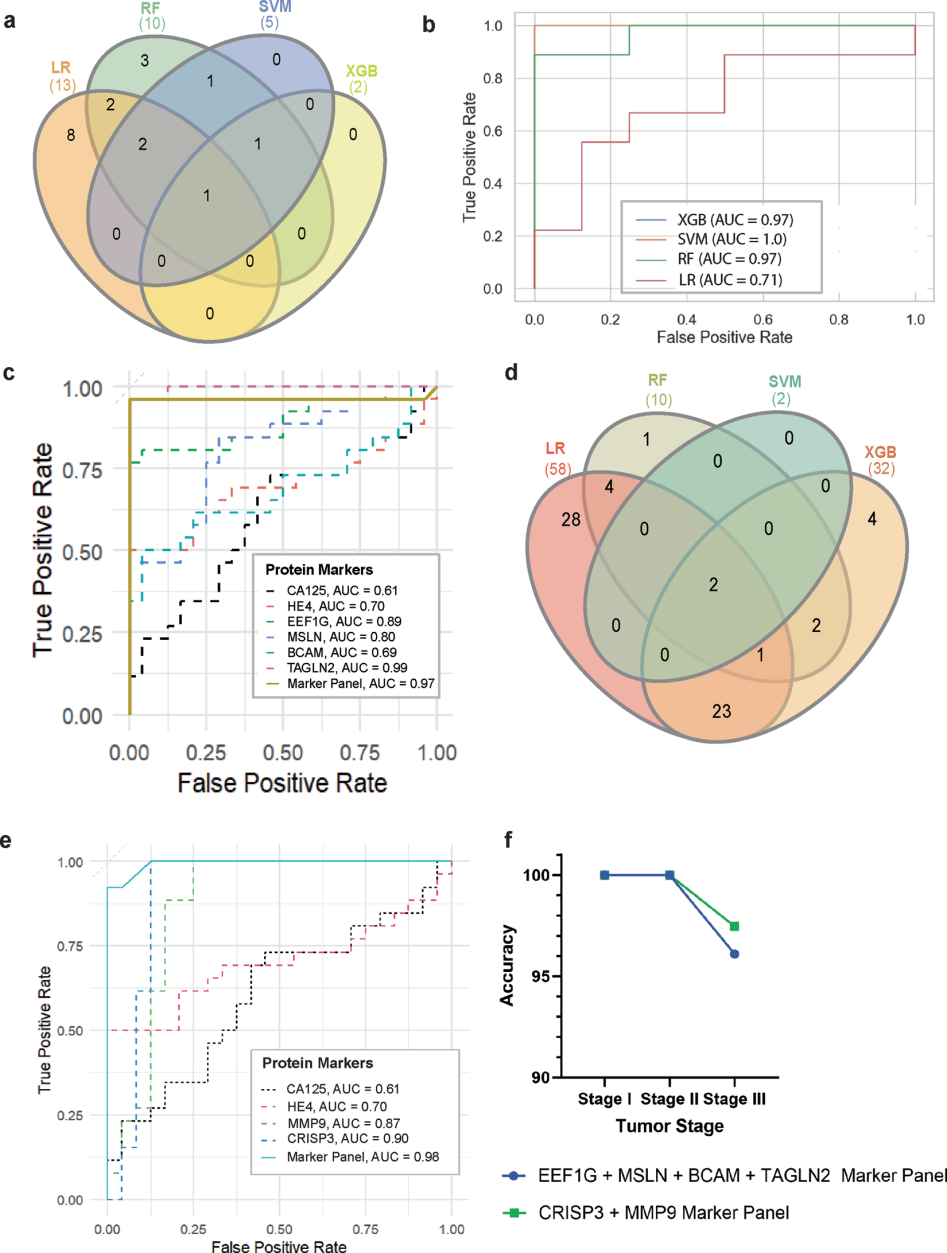
Machine learning based feature extraction. (**a**) Venn diagram of the selected features by the classifiers: LR, RF, SVM, XGB for the CDPs in cluster 1. (**b**) The receiver operating graph of the commonly selected features by the classifiers; RF, SVC, XGB, LR on the hold-out test dataset for cluster 1. The area under the curve (AUC) is calculated for each classifier. (**c**) Receiver operator graph with the associated AUC for the cluster 1 shortlisted markers EEF1G, MSLN, BCAM, TAGLN2 individually and as a signature and the currently utilized ovarian cancer markers CA125, HE4. (**d**) Venn diagram of the selected features by the classifiers: LR, RF, SVM, XGB for the CDPs in cluster 2. (**e**) Receiver operator graph with the associated AUC for the cluster 2 shortlisted markers CRISP3, MMP9 individually and as a signature and the currently utilized ovarian cancer markers CA125, HE4. (**f**) Classification accuracy variation across the histological statues of HGSOC tumor on the discovery test cohort of EEF1G + MSLN + BCAM + TAGLN2 marker panel and CRISP3 + MMP9 (Venn diagrams were generated by InteractiVenn^69^).

To identify discriminative protein markers from CDPs, a comprehensive ML exercise was carried out on the serum dataset (Supplementary Fig. 3a). This exercise comprised two steps - feature selection and classifier development. Recursive feature selection^23^ (RFS) and sequential feature selection^24^ (SFS) methods were applied to CDPs belonging to clusters 1 and 2 using 20% of patient samples from the serum dataset. This ML exercise was coupled with 5-fold cross-validation with logistic regression (LR), support vector machine (SVM), random forest (RF), and extreme gradient boosting algorithms (XGB) as classifiers. RFS (Supplementary Fig. 1b) resulted in selecting 13, 10, 5, 2 markers from CDP cluster 1 (Fig. 3a, Supplementary Table 3) and 58, 10, 2, 32 markers from the CDP cluster 2 (Fig. 3d, Supplementary Table 3) based on classifier performance evaluated by cross-validation accuracy (AUC) (Supplementary Fig. 3b). Markers selected by at least two classifiers were added into the shortlisted marker panel. This resulted in 10 serum markers for CDP cluster 1 and 32 protein serum markers for CDP cluster 2 in segregating HGSOC from healthy with RFS. To select the optimal number of biomarkers, SFS was applied with forward and backward directions, using the F1 score as the scoring matric. This resulted in shortlisting EEF1G + MSLN + BCAM + TAGLN2 as the most distinctive markers differentiating between HGSOC and healthy cohort with 0.97 AUC for cluster 1 (Fig. 3b,c) and selecting CRISP3 + MMP9 with 0.98 in AUC for cluster 2 (Fig. 3e).

To evaluate the classification performance of these marker panels two ML models were built with 60% of patient samples from the serum dataset using the shortlisted markers as the features (Supplementary Fig. 3c). We compared 4 ML classifiers with the above marker panels and determined XGB models as the final classifiers for their overall superior performance, interpretability, and low risk of data overfitting (Fig. 3b). Nevertheless, predictive markers’ performance was consistent across classification algorithms tested with XGB, SVM, RF, and LR (Fig. 3b). The hold-out dataset comprised of 20% of the patients’ samples in tissue and serum datasets were subjected to perform the classifier testing (Supplementary Fig. 4a,c). The ROC analysis of the novel marker panel EEF1G + MSLN + BCAM + TAGLN2 demonstrated a 59.1% increase in AUC compared to the most widely used clinical marker, CA125, and a 38.6% increase compared to HE4 (Fig. 3c). Similarly, the marker pair CRISP3 + MMP9 showcased a 60.7% increase in AUC compared to CA125 and a 40.0% increase compared to HE4 (Fig. 3e). Furthermore, when these results were categorized by the tumor stage, the classifiers were able to correctly classify lower stages of Stage I, II and also Stage IV for both tissue and serum test cohorts (Fig. 3f).

### Assessing performances of new biomarkers

**Fig. 4:**
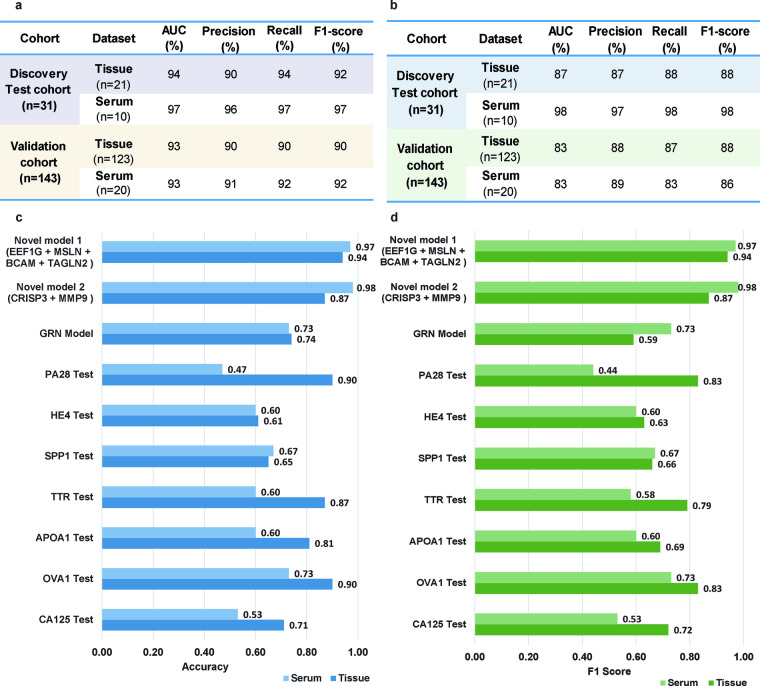
Novel HGSOC marker signature verification. (**a**) Table with machine learning classifier performance results on the hold-out test cohorts and independent verification cohorts for marker panel EEF1G + MSLN + BCAM + TAGLN2 and marker pair CRISP3 + MMP9. (**b,c**) Balanced accuracy, **(d)** F1 score of the classifier built on the novel marker panel, marker pair, best in class markers utilized currently in practice GRN, PA28, HE4, SPP1, TTR, APOA1, OVA1, and CA125 in both tissue and serum verification cohorts.

We carried out performance verification of identified marker panels using two publicly available serum and tissue proteomics datasets (Supplementary Fig. 3c). The serum dataset^25^ comprised of 10 individuals diagnosed with HGSOC and 10 healthy samples. The proteomics models correctly excluded HGSOC samples from the healthy with an AUC of 93% and 92% F-score for the EEF1G + MSLN + BCAM + TAGLN2 marker panel (Fig. 4a) and an AUC of 83%, and 86% F1 score CRISP3 + MMP9 marker pair (Fig. 4b). A tissue based HGSOC verification cohort consisting 103 tumor samples and 10 healthy samples (https://pdc.cancer.gov/pdc/study/PDC000113) was employed. The proteomics model with EEF1G + MSLN + BCAM + TAGLN2 marker panel in tissue yielded 93% in AUC and 90% F-score (Fig. 4a) while CRISP3 + MMP9 marker pair yielded 83% in AUC and 88% in F1-score (Fig. 4b). Since these datasets were not well balanced in terms of the tumor and healthy sample proportions, the balanced classifier performance, and weighted evaluation matrices were utilized to assess the linear classifiers’ performance. For the tree-based classifiers, weighted subsampling with balanced class proportions was computed (Supplementary Fig. 4b,d).

Additionally, we compared our proteomics classifier model against seven known OC biomarkers CA125^26^, HE4^18^, APOA1^27^, TTR^27^, SPP1^28^, PA28^18^, and GRN^29^ and a FDA approved biomarker test OVA1^30^. Both of our novel proteomics biomarker panels exhibited the most promising balanced accuracies (Fig. 4c) and F1 scores (Fig. 4d) in both serum and tissue. It was notable that the PA28 test (90% in AUC) and OVA1 test (90% in AUC) showed significantly similar and slightly better performance to our proposed marker panels in serum (87% in AUC). However, their performance was lacking in tissue (47% and 73% in AUC for PA28 and OVA1 respectively), whereas our panels demonstrated AUCs of 94% in the EEF1G + MSLN + BCAM + TAGLN2 panel and 87% in the CRISP3 + MMP9 panel. Moreover, the survival analysis of the identified markers revealed increased expression of these markers in the transcriptome leads to poor survival in all six proteins (Supplementary Fig. 5). We believe that the efficacy of these new biomarkers, exhibiting high performance in both tissue and serum, will not only aid in HGSOC identification but also contribute to prognostic evaluations.

## Discussion

We exploited advanced analytical methodologies, predominantly a fusion of ML models, to systematically extract new biological insights and novel biomarkers for HGSOC utilizing publicly available serum and ovary tissue proteomic datasets. Our pipeline provides extracting candidate markers through systematic steps, which are independent of protein quantification methods and sample types. For example, in this study, we used label-based tissue and label-free serum datasets as inputs for the analysis that allowed revealing common features for both data and sample types. Using this systematic integrated process, we identified novel biological insights into ovarian cancer and an outperforming discriminative serum biomarker pair and panel for HGSOC with clinical diagnostic potential. We verified the performances of these markers using publicly available different tissue and serum proteomics datasets, which corroborated the model’s performance in identifying early-stage HGSOC, as determined by AUC.

Our functional enrichment analysis of CDPs revealed interesting observations pertaining to disease processes. For example, an inversely correlated CDPs cluster with up in serum and down in tissue consists of several ECM players. They include a variety of collagen matrix elements (COL6A2, COL6A), fibrous (TNXB), and glycol proteins (LUM). TNXB, exhibiting the largest molecular proportions among the tenascins, shows a high correlation with CA125 and has been proposed as a potential biomarker for early ovarian cancer diagnosis^31,32^. Nevertheless, its contribution to the ECM in HGSOC has not yet been explored. LUM is another ECM protein associated with cell adhesion and migration regulation in HGSOC homeostasis^33,34^. These dysregulations indicate potential avenues for further exploration into the mechanisms underlying HGSOC.

In this study, we confidently identified the marker combination of EEF1G + MSLN + BCAM + TAGLN2 which showed upregulation in both serum and tissue an outperforming marker panel against currently available markers for ovarian cancer. These markers are known to play important roles in cancer progression^35^ (Supplementary Fig. 6). For example, EEF1G belongs to the eukaryotic translation elongation factor family that plays a central role in the elongation step of translation but is often altered in many cancer types including multiple myeloma^36^, glioblastoma^37^, bone osteosarcoma and prostate carcinoma^38^. A study shows that higher expression of EEF1G predicted better overall survival and progression-free survival in OC patients^39^. MSLN is identified as a critical player in regulating ovarian cancer pathophysiology through IL-6/STAT3 signaling^19^ while correlating with immune infiltration and chemoresistance as a prognostic biomarker in ovarian cancer^40^. Moreover, MSLN’s impact on the ovarian cancer microenvironment was known to be involved in cell survival, proliferation, tumor progression, and adherence^41^. MSLN has been shown to bind to CA-125 and is thought to play a role in the peritoneal diffusion of ovarian tumor cells^41^. A recent study reports that the dysregulation of MSLN in serum and tissue acts as a promising diagnostic biomarker for gastric cancer^42^. Basal cell adhesion molecule (BCAM) is another important protein reported in ovarian cancer playing a key role in the metastasis process^43,44^ and immune suppression^45^. The recurrent BCAM-AKT2 fusion gene leads to activated AKT2 function kinase in HGSOC compared to the healthy^46^. TAGLN2 overexpression is also associated with the malignant transformation of cancer, such as resistance, metastasis, and invasion contributing as a candidate biomarker for diagnosis, treatment, and prognosis of cancer^47^. TAGLN2 is found to be an important protein in the ovarian cancer microenvironment by cytoskeletal organization^40^. It has been reported as a serum extracellular vesicle circulating biomarker in adenomyosis^48^ and tumor promotor in papillary thyroid carcinoma via the Rap1/PI3K/AKT axis^17^. Here, our state-of-the-art computational analysis suggests that combination of these four makers can serve as a specific marker panel for the diagnosis of HGSOC.

Of note, we further observed that the marker pair CRISP3 and MMP9 exhibited inverse dysregulation and achieved the highest AUC. These markers also provide prognostic insights and clues to unravel the defense mechanisms against HGSOC. CRISP3 was shown to be elevated in prostate tumors and linked to cancer progression from primary to metastatic prostate cancer^49,50^. Elevated CRISP3 in serum level is associated with poor treatment outcomes and also plays a role in predicting responses to treatments such as androgen deprivation therapy (ADT) and chemotherapy^51,52^. Downregulated CRISP3 has been shown in the breast^53^, cervical^54^, and ovarian cancer tissues^55,56^. In breast cancer, low levels of CRISP3 in tissue are correlated with poor survival rates^53^, whereas in ovarian cancer, increased expression of CRISP3 in serum is associated with HGSOC and poorer survival outcomes (Fig. 4d). Given these findings and the commonalities of its association with various cancer types, CRISP3 is a functionally relevant potential member of the new HGSOC marker panel. MMP9 is an enzyme involved in breaking down components of the ECM^57^, playing a role in various physiological and pathological processes, including cancer^58^. It is often overexpressed in multiple cancer types, promoting cancer cell invasion and metastasis by disrupting ECM, enabling cancer cells to spread to distant locations^59^. In ovarian cancer, MMP9 has been found to be associated with tumor invasion, metastasis, and angiogenesis^60,61^. Elevated MMP9 levels in HGSOC serum are linked to advanced disease stages^62^ and a poor prognosis (Supplementary Fig. 5e). MMP9 also creates an immune-suppressive environment in ovarian tumors^63^, hindering the body’s defense against cancer cells^64^. Our study’s findings on MMP9’s co-dysregulation in both tumor and serum, along with its known associations in ovarian cancer progression, suggest its potential as a diagnostic marker for HGSOC. Understanding how its dysregulation affects ECM remodeling may provide valuable insights for potential therapeutic approaches.

This study’s strengths lie in its utilization of publicly available, high quality MS-based data, which includes ovary biopsy and serum samples, providing valuable insights into proteomic-level co-dysregulation in an unbiased fashion. Moreover, both the derivation and verification cohorts encompassed a diverse patient population from around the world, thereby representing the complete spectrum of individuals with HGSOC prior to undergoing chemotherapy. This ML approach utilized here was crafted to increase the disease specificity of the markers. Notably, our proteomic HGSOC models exhibited the capability to distinguish HGSOC cases from a subset of healthy individuals confidently and accurately diagnosed participants in the two independent verification cohorts. One limitation of this work is the lack of more independent validation datasets with large sample sizes. Hence, our identified marker panels together with other known markers should be rigorously validated using clinical diagnostic compatible approaches such as enzyme-linked immunosorbent assay (ELISA) in well-defined large patient cohorts including other ovarian and cancer types.

In summary, by leveraging the strategized ML capabilities, this study unveils a panel of high-performing novel biomarkers with diagnostic potential for identification of HGSOC and functional associations, which shed light on HGSOC clinical management and novel therapeutics intervention of this aggressive cancer type.

## Methods

### Data sources

The publicly available Ovarian Cancer Confirmatory Study Proteomic Dataset (PDC000114) in the CPTAC data portal (https://proteomic.datacommons.cancer.gov/pdc/) was used as the tissued-based diagnostic dataset^9^. This dataset comprises 83 early-stage HGSOC samples and 20 healthy controls processed in the Pacific Northwest National Laboratory. It encompassed 1 of Stage I, 6 of Stage II, 64 of Stage III, and 12 of Stage IV samples. This dataset serves as a complementary dataset for the comprehensive proteogenomic ovarian cancer categorization with healthy samples with the identification of 8703 quantified protein groups using tandem mass tags isobaric labeling-based mass spectrometry analysis^65^. The serum proteomics dataset^10^ comprised 26 HGSOC cases with 3 Stage I, 3 Stage II, and 20 Stage III samples and 24 healthy controls. It consists of 1,847 quantified proteins across all samples with label-free quantification-based mass spectrometry analysis.

For performance evaluation analysis, two published serum and tissue datasets, different from the above-mentioned datasets were used. The serum verification cohort^25^ comprised 20 clinical samples with 10 individuals with HGSOC with 5 of each Stage III and Stage IV samples and 10 healthy controls. The tissue verification cohort (https://pdc.cancer.gov/pdc/study/PDC000113) included 103 tissue samples with 2 of Stage I, 4 of Stage II, 82 of Stage III, and 15 of Stage IV samples and 10 healthy controls.

### Integration of ovary tissue and serum proteomics

Supplementary Table 1 provides the list of co-dysregulated proteins (CDPs) along with respective log fold change (LFC), and FDR-corrected p-values generated from the t-test with original datasets. The LFC for a protein is defined as the log ratio between “Tumor” samples into “Healthy” samples. LFC-based similarity analysis was conducted to evaluate the dysregulations between ovary biopsy and serum with CDPs by plotting the serum LFC as the function of ovary tissue LFC.

### Functional enrichment analysis

Ontology enrichment analysis of the CDPs was conducted using the David Bioinformatics Functional Annotation Platform^66^ available at https://david.ncifcrf.gov/home.jsp with default settings. Supplementary Table 2 includes the list of significantly enriched pathway terms^67^ and associated proteins. The gene ontologies were considered for biological processes, and molecular functions.

### ML model construction

Three steps starting from feature selection, classifier development, evaluation, and verification were included in the ML framework for this study (Fig. 1). A serum protein matrix was utilized as the input, with each row representing a patient sample and each column representing a protein involved in the task. For binary classification, HGSOC samples were labeled as 1 and healthy controls as 0. The serum dataset was divided into three parts: feature selection 10 samples (20% of the dataset), classifier creation 30 samples (60% of the dataset), and testing 10 samples (20% of the dataset). RFS and SFS were utilized as the feature selection methods. RFS is a method for feature selection that iteratively fits a model and removes the least important feature(s) until the optimal number of features for the classification task is reached (Refer to Supplementary Figure S3B for detailed steps). The model-ranked features were based on their importance scores, aiming to eliminate interdependencies and collinearity. Since RFS requires a set of features to retain at the beginning, determining the optimal count beforehand is challenging. Cross-validation was employed with RFS to evaluate various feature subsets and identify the most effective set of features. RFS was executed using yellowbrick.model_selection.RFECV (yellowbrick library version 1.5), employing LR with sklearn.linear_model.SGDClassifier (scikit-learn library version 1.3.0), SVM with sklearn.svm.SVC, RF with sklearn.ensemble. RandomForestClassifier, and XGB with xgboost.XGBClassifier (xgboost library version 1.7.6) as the estimators, all with default parameters. Subsequently, the features commonly identified by at least two of these estimators were selected to perform SFS. SFS operates by either adding (forward selection) or removing (backward selection) features to create a feature subset in a greedy manner. At each step, the estimator selects the optimal feature to include or exclude, based on the cross-validation score. SFS was implemented using sklearn.feature_selection.SequentialFeatureSelector with the aforementioned estimators and default settings.

The second step of the pipeline is classifier development. For this, estimators including LR, SVM, RF, and XGB algorithms were employed. LR was implemented using sklearn.linear_model.SGDClassifier with default parameters and optimized settings, including 1000 iterations (epochs), an error tolerance of 10^−5^, and a regularization term multiplier set to 0.5. SVM utilized the linear kernel with default parameters from sklearn.svm.SVC. The RF model employed sklearn.ensemble.RandomForestClassifier with a maximum tree depth of 5 and 100 estimators. The XGB model utilized xgboost.XGBClassifier, with parameters set to a learning rate of 0.2, 1000 estimators, a maximum tree depth of 5, and a subsampling proportion of 0.8 during training.

To prevent overfitting of the models due to limited sample sizes, comprehensive cross-validation was performed. The sklearn.model_selection.RepeatedStratifiedKFold was utilized to implement the fivefold cross-validation procedure with repetition of three times. Within each fivefold cross-validation iteration, the training dataset was divided into five smaller sets (folds 1–5). These sets were further split into train–test pairs, with each iteration serving as a test set. LR, SVM, RF, and XGB models were independently trained on the training sets and evaluated their performance on the corresponding test sets. This procedure was repeated three times with different randomizations, resulting in 30 train–test pairs for each model (totaling 120 trained models). The average performance of each model across the 30 test sets was then computed and presented. In the cross-validation procedure, the serum-based model had 24/6 for train/test splits. To assess the performance of the identified markers in tissue, a tissue-based machine learning model utilizing XGBoost was constructed as described above.

To address the class imbalances of the training datasets, two customizations of balanced classifiers were developed. The first approach named ‘dictionary balanced’ was used to calculate the class weight as follows.

$$Class\,weight\,for\,class\,{C}_{i}=\frac{Total\,number\,of\,samples-Number\,of\,samples\,in\,{C}_{i}}{Number\,of\,samples\,in\,{C}_{i}}$$

The second method ‘package balanced’ computes the weight vector for a class using the class labels directly to automatically adjust weights inversely proportional to the class frequencies as follows.

$$Weight\,vector\,for\,class\,{C}_{i}=\frac{Total\,number\,of\,samples}{Number\,of\,classes\ast Class\,frequency\,vector\,of\,{C}_{i}}$$

The dictionary balanced-based classifiers were implemented for LR, SVM, RF, and XGB, however, the package balanced was only supported for LR, RF, and XGB. Moreover, another balancing classifier creation is only applicable for RF and XGB which is similar to ‘package balanced’ named ‘subsample balanced’ except that weights were computed based on the bootstrap sample for every tree grown were employed.

To assess the performance of the classifier models with the test data and independent verification cohorts, the metrics of the AUC using precision (precision_macro), recall (recall_macro), F1-score (f1_macro) which outputs the average metric value without considering the proportion for each label in the dataset were used. Since the testing and verification datasets were class imbalanced, weighted metrics for F1-score (f1_weighted), precision (precision_weighted), and recall (recall_weighted) where the class proportions are reflected as the weights were employed.

### Survival analysis

Survival analysis of the shortlisted markers was conducted using the KMPlot web application^68^ available at https://kmplot.com/analysis/index.php with default settings.

### Supplementary information

Supplementary Table 1

Supplementary Table 2

Supplementary Table 3

Supplementary Information

## Data Availability

The tissue proteomics data supporting the current work can be accessed at CPTAC Ovarian cancer repository (https://proteomic.datacommons.cancer.gov/pdc) with dataset ID PDC000114^9^ and the serum proteomics data was obtained from the supporting information of Huh, *et al*.^10^ study https://pubs.acs.org/doi/10.1021/acs.jproteome.2c00218, pr2c00218_si_002.xlsx. The data used for performance evaluation in this article were obtained from Ahn *et al*.^25^ 10.3390/cancers12113447 for the serum verification cohort and tissue cohort in the CPTAC ovarian cancer repository with dataset ID PDC000113. The differential expression analysis of the CDPs was uploaded to Supplementary Table 1. The KEGG pathway enrichment expression analysis results of HGSOC tissue samples are recorded in Supplementary Table 2. The ML-based shortlisted markers are listed in Supplementary Table 3.

## Code availability

The analysis methodology associated with this article is available on GitHub (https://github.com/kts-desilva/UMAI).
