# Supplementary material for: Machine Learning-Enhanced Extraction of Biomarkers for High-Grade Serous Ovarian Cancer from Proteomics Data
Source: Sci Data. 2024 Jun 25;11:685. doi: 10.1038/s41597-024-03536-1 (PMC11199488; doi:10.1038/s41597-024-03536-1)
Supplement: Supplementary file 4 — Supplementary Information [file 41597_2024_3536_MOESM4_ESM.pdf]

Supplementary Information

Machine Learning-Enhanced Extraction of Biomarkers for High-Grade Serous Ovarian Cancer from Proteomics Data

Senuri De Silva[1,2], Asfa Alli-Shaik[1], Jayantha Gunaratne[1,2,*]

[1] Institute of Molecular and Cell Biology (IMCB), Agency for Science, Technology and Research (A*STAR), Singapore 138673

[2] Yong Loo Lin School of Medicine, National University of Singapore, Singapore 117594

* Corresponding author: Jayantha Gunaratne

This supplementary information includes:

Supplementary Figure 1 —— 2

Supplementary Figure 2 —— 3

Supplementary Figure 3 —— 4

Supplementary Figure 4 —— 6

Supplementary Figure 5 —— 7

Supplementary Figure 6 —— 8

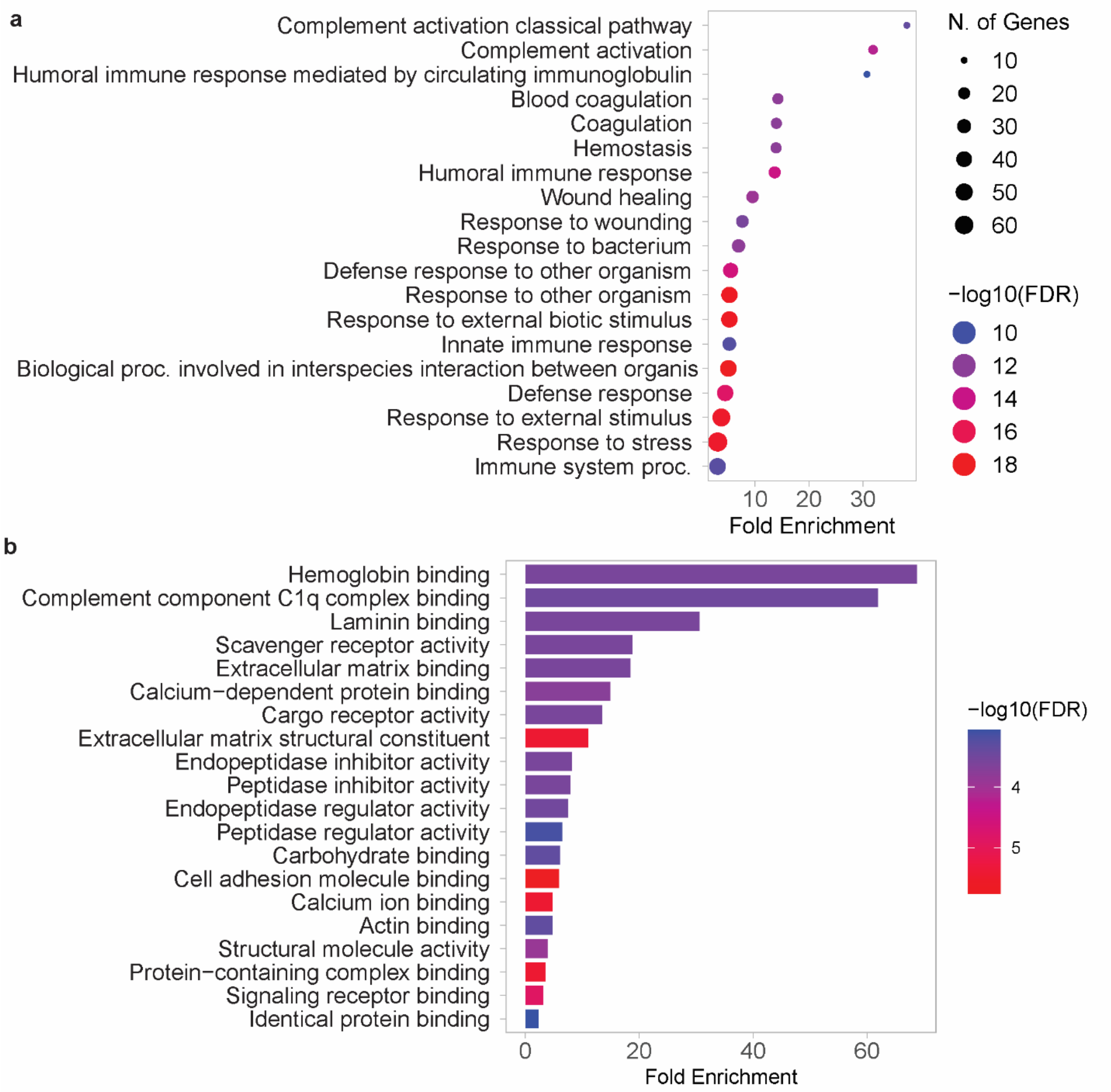

Supplementary Figure 1. Functional enrichment of co-dysregulated proteins. a. The top biological processes enriched on the co-dysregulated proteins (CDPs) with GO terms (v2023.2.Hs) where horizontal axis presents the fold enrichment, and the number genes are represented by the size of the dot and the color represent the FDR corrected log fold change with log10 conversion. b. The top molecular functions enriched on the dysregulated proteins with GO terms (v2023.2.Hs) CDPs and the length of the bar represents the fold enrichment, and the bar color represents the FDR corrected p-value for the enrichment.

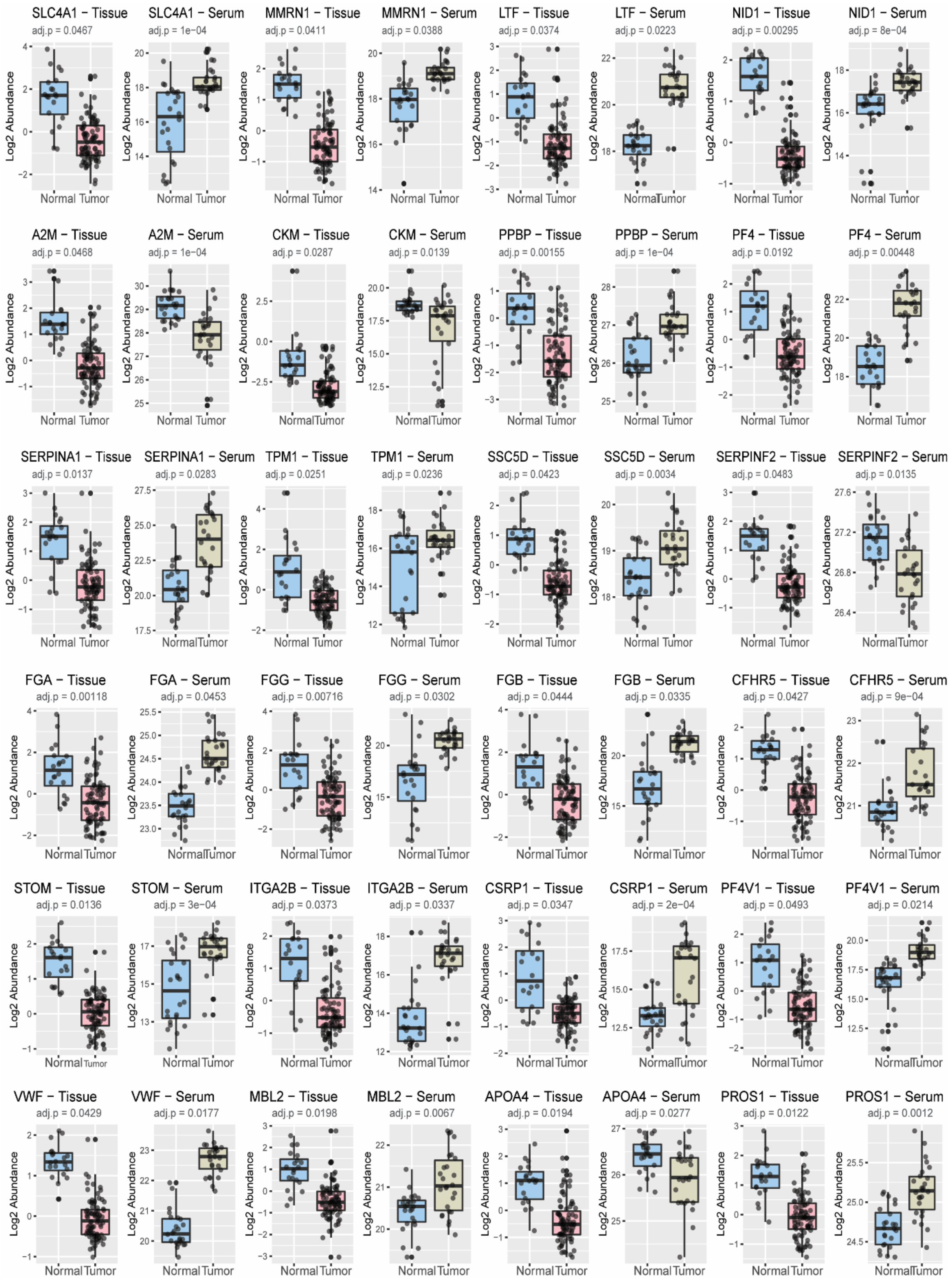

Supplementary Figure 2. Co-dysregulated proteins in tissue and serum. Boxplots displaying the top 7-30 commonly dysregulated proteins in ovarian tissue (left) and serum (right), depicting healthy controls (blue) versus tumor samples (pink for tissue and yellow for serum). The normalized protein log2 abundance and adjusted p-values from the t-test are shown.

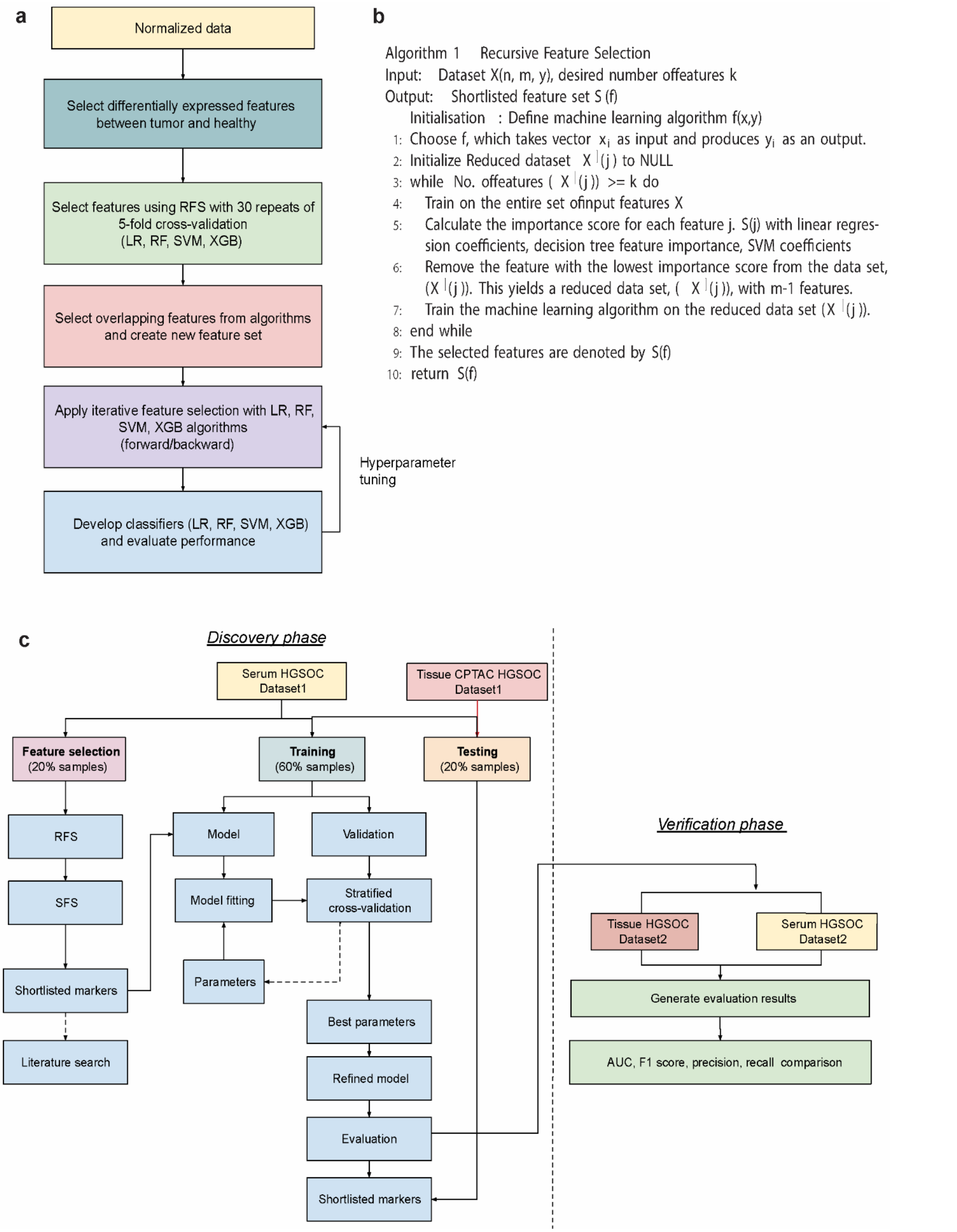

Supplementary Figure 3. Machine learning based feature extraction workflows. a. The workflow for machine learning-based feature extraction begins with differential expression analysis, followed by Recursive Feature Selection (RFS) using Linear Regression (LR), Random Forest (RF), Support Vector

Machine (SVM), and XGBoost (XGB), with subsequent extraction of overlapping features. Next, iterative feature selection is performed using LR, RF, SVM, and XGB with both forward and backward selection methods to finalize discriminative markers. Classifiers are then developed, and hyperparameter tuning is conducted using LR, RF, SVM, and XGB to evaluate the shortlisted features. b. The step-by-step decomposition of the RFS algorithm using a normalized protein matrix where samples are represented as rows and proteins as columns. The output showcases the shortlisted features identified by the RFS algorithm. c. The discovery and verification strategy decomposition of the study, detailing the utilization of datasets in both the discovery and verification phases, along with each processing step involved in the verification process. The discovery phase comprised of feature selection with RFS, SFS, model training and model testing. The verification phase involves in utilizing two public datasets on the derived markers from the discovery phase to obtain evaluation results.

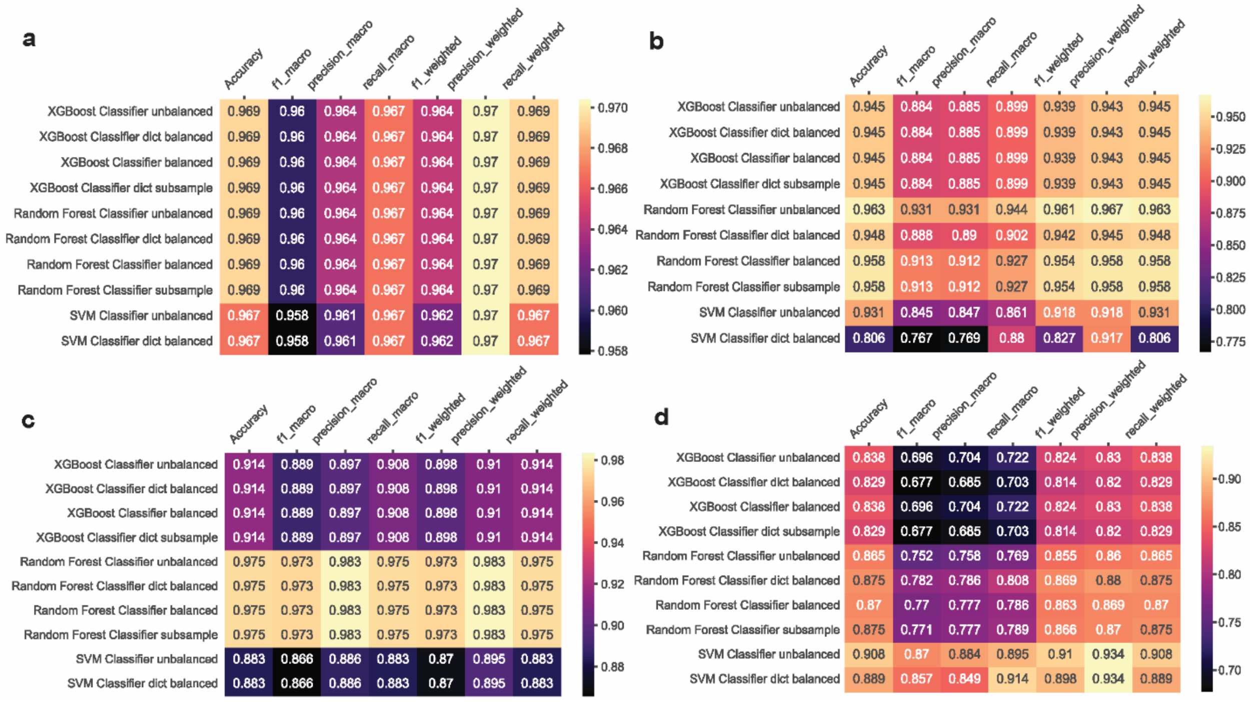

Supplementary Figure 4. Machine learning classifier evaluation. a, b. The accuracy, F1 score, precision, recall without considering the class imbalanced (f1_macro, precision_macro, recall_macro), and F1 score, precision, recall computed considering the class imbalances (f1_weighted, precision_weighted, recall_weighted) values of the classifiers XGB, RF, SVM, LR created with all training samples (unbalanced), weights defined for each class inversely based on the number of samples in each class (dict balanced), weights defined by the number of samples of particular class divided by the number of classes multiplied by the class assignment vector (balanced), for tree based classifiers XGBoost, RandomForest subsampling is performed having equal sample sizes in each round of tree creation (subsample) on the serum verification cohort (a) and the tissue verification cohort (b) for marker panel EEF1G + MSLN + BCAM + TAGLN2 and marker pair CRISP3 + MMP9 on the serum verification cohort (c) and tissue verification cohort (d).

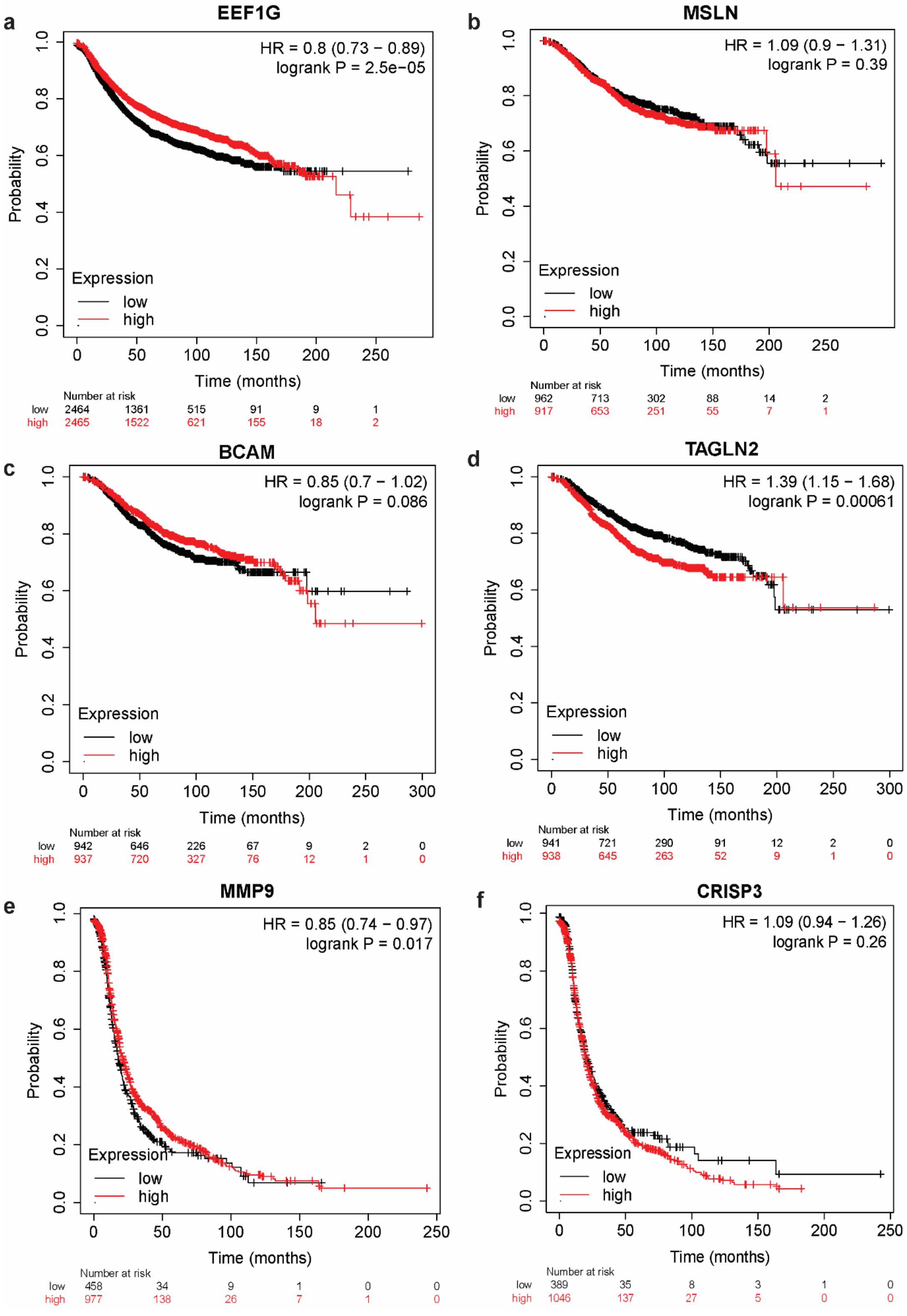

Supplementary Figure 5. Survival analysis of the signatures. Survival curves derived from KMPlot[68] application on the shortlisted markers in the panels; EEF1G (a), MSLN (b), BCAM (c), TAGLN2 (d), CRISP3 (e), MMP9 (f).

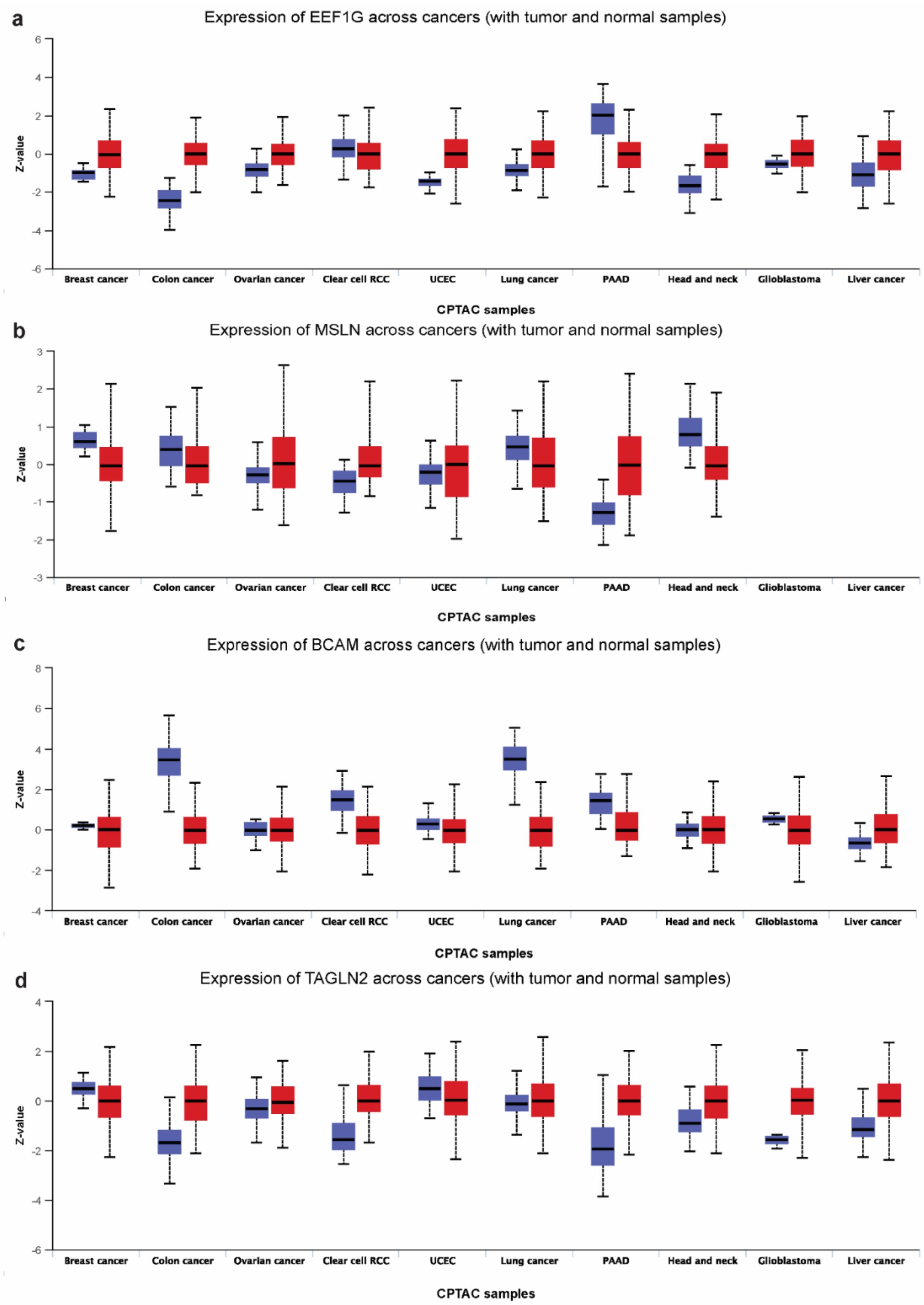

Supplementary Figure 6. Expression of the identified signatures in other cancer types. The signature expressions on multiple cancer types based on publicly available CPTAC data[35] including breast cancer, colon cancer, clear cell renal cancer (RCC), uterine corpus endometrial carcinoma (UCEC), lung cancer, pancreatic adenocarcinoma (PAAD), head and neck carcinoma, glioblastoma and liver cancer on the signatures EEF1G (a), MSLN (b), BCAM (c), TAGLN2 (d).
